# Practical Text-Book of Midwifery for Nurses

**Published:** 1904-12

**Authors:** 


**Practical Text-book of Midwifery for Nurses.** By Robert Jardine, M.D. Second Edition. Pp. xv., 268. Edinburgh: William F. Clay. 1903.

This is a reprint of nursing lectures given in the Glasgow Maternity Hospital. It has only 250 small pages, and therefore many subjects are but lightly dealt with. The mechanism of labour is essential to the understanding of any case, and therefore it should be most carefully treated in any book intended as a guide to practice. Only a few pages are devoted to this.

Some subjects are very curtly dismissed. For instance, "In the concealed form of accidental hemorrhage, the only hope lies in forcible dilatation and delivery, or by doing a Porro's operation." Surely rupture of the membranes comes before these.

The diseases of the child, so important to nurses, are little more than tabulated. There is more said on syphilis than indigestion.

Allowing for these defects, mostly due to want of space, the book is readable, and may be useful to beginners.

